# Association between NMDA gene polymorphism (rs4880213) and GRIN2B blood serum levels in thyroid pathology patients

**DOI:** 10.25122/jml-2021-0372

**Published:** 2022-01

**Authors:** Iryna Ivanivna Kamyshna, Larysa Borysivna Pavlovych, Aleksandr Mychailovich Kamyshnyi

**Affiliations:** 1. Department of Medical Rehabilitation, Ivan Horbachevsky Ternopil National Medical University, Ternopil, Ukraine; 2. Department of Clinical Immunology, Allergology and Endocrinology, Bukovinian State Medical University, Chernivtsi, Ukraine; 3. Department of Microbiology, Virology, and Immunology, Ivan Horbachevsky Ternopil National Medical University, Ternopil, Ukraine

**Keywords:** GRIN2B, NMDA, hypothyroidism, autoimmune thyroiditis, GRIN2B – N-Methyl-D-Aspartate 2B, NMDA – N-methyl-d-aspartate, NMDAR – N-methyl-d-aspartate receptors, SNPs – Specific single nucleotide polymorphisms, T4 – Thyroid hormones L-thyroxine, T3 – 3,3,5-triiodothyronine, GluR – glutamate receptor, AIT – autoimmune thyroiditis, PO – postoperative hypothyroidism, anti-Tg – anti-thyroglobulin, anti-TPO – anti-thyroid peroxidase

## Abstract

The article discusses a new hypothesis that autoimmune diseases of the thyroid gland can lead to depression and neurological complications. It is believed that the neuronal N-methyl-D-aspartate receptor plays a significant role in depression pathophysiology and neurological and mental diseases, respectively. The study involved 153 patients with various forms of thyroid pathology. GRIN2B levels in the sera of the patients and healthy individuals were quantified using enzyme-linked immunosorbent assay with highly sensitive Human GRIN2B (Glutamate Receptor, Ionotropic, N-Methyl-D-Aspartate 2B) ELISA Kit. Genotyping of the glutamate ionotropic receptor NMDA type subunit 1, GRIN1 (rs4880213) gene polymorphism. The CT genotype of the NMDA gene (rs4880213) was predominant in the surveyed population. The C allele of the NMDA gene was more frequent than the T allele among patients with thyroid disease. GRIN2B levels were significantly decreased in patients with postoperative hypothyroidism 3.45 times, and in patients with AIT-induced hypothyroidism, there was a probable increase in GRIN2B levels by 1.58 times compared with controls. GRIN2B levels were significantly different in patients of different groups depending on thyroid pathology. Our study showed direct close correlation (r=0.635) between GRIN2B and anti-TPO levels (p<0.001), a significant direct close correlation (r=0.527) between GRIN2B and anti-TG levels in the blood (p<0.001). Our results allow us to consider the GRIN2B level as an important prognostic minimally invasive marker of neurological complications in endocrine pathology.

## Introduction

Autoimmune diseases of the thyroid gland impact the transcriptional activity of regulator genes of neurogenesis and neurotrophins and can lead to depression and neurological complications. In addition, it is believed that the neuronal N-methyl-D-aspartate receptor (NMDAR) plays a significant role in depression pathophysiology and neurological and mental diseases, respectively [1]. The composition of subunits can influence several NMDAR physiological and pharmacological characteristics [2, 3]. Specific single nucleotide polymorphisms (SNPs) in the genes of NMDAR subunits have been detected to influence NMDAR signaling pathways in the pathogenesis of different types of mood disorders by changing their expression, distribution, and activity. Thyroid hormones L-thyroxine (T4) and 3,3,5-triiodothyronine (T3) are critical for healthy brain development and proper function [4]. Current data suggest that glutamate-mediated neurotransmission may be an essential target for thyroid hormones [5]. In animals with hypothyroidism, there is a decrease in glutamate synthesis with a subsequent reduction in the release of vesicular glutamate in the CA3-hippocampus [6]. A pronounced decrease in glutamate content was also found in the hippocampus of patients with hypothyroidism [7]. In rodents, adult hypothyroidism reduced the expression in the hippocampus of mRNA encoding the NR1 subunit of the N-methyl-D-aspartate receptor complex (NMDA) and improved NR2B expression [8]. In contrast, hyperthyroidism selectively reduces NR2B mRNA expression in the dorsal hippocampus [9].

Only several studies have concentrated their attention on GRIN1 rs4880213 and the pathophysiology of human diseases resulting from it. However, two studies reported that patients having GRIN1 rs4880213 C/C variation showed a more serious disability and decreased NMDAR-mediated cortical response than subjects with C/T or T/T variations [10, 11]. In addition, variants in the NMDAR 2B subunit gene (GRIN2B) have been linked to schizophrenia, mental illnesses, and brain plasticity [12, 13]. Hashimoto thyroiditis is reported as one of the autoimmune diseases related to mental disorders. Some studies have demonstrated a significant prevalence of antibodies to the N-terminus of glutamate receptor (GluR) subunits of the N-methyl-D-aspartate (NMDA) type (GluN1-NT and GluN2B-NT2) among subjects suffering from psychiatric disorders with anti-thyroid antibodies [14]. Previously, we reported autoimmune thyroiditis (AIT) and hypothyroidism to affect transcription of the genes implicated in neurogenesis, the transmission of nerve impulses, and the regulation of the cell cycle [15–19]. In the present study, we examined the NMDA gene polymorphism (rs4880213) and the GRIN2B blood serum levels in patients with thyroid pathology in the population of Western Ukraine.

## Material and Methods

Our study included 153 patients with different types of thyroid disorders. The subjects were distributed into three groups. Group 1 (n=16) included patients experiencing postoperative hypothyroidism (PO); group 2 (n=65) included patients suffering from hypothyroidism resulting from autoimmune thyroiditis (AIT), and group 3 (n=72) included patients with both AIT and raised serum antibodies anti-thyroglobulin (anti-Tg) and anti-thyroid peroxidase (anti-TPO). Twenty-five healthy subjects were randomly recruited as a control group without age and gender matching. The clinical and biochemical features of the subjects are displayed in Table 1.

**Table 1.** Clinical and biochemical characteristics of the subjects.

	**Control** **group** **(n=25)**	**Patients with postoperative hypothyroidism (PO, n=16)**	**Patients with AIT-induced hypothyroidism** **(AIT with hypothyroidism, n=65)**	**Patients with AIT and elevated anti-Tg and anti-TPO antibodies** **(AIT, n=72)**
**Age (years)**	46.08±14.58	47.30±12.27	46.72±15.49	45.02±13.65
**fT4 (pmol/L)**	8.91±0.97	3.44±0.31	4.13±0.52	8.51±0.82
**TSH (mIU/mL)**	2.67±0.52	8.61±0.84	7.09±0.50	2.38±0.62
**anti-TPO (IU/mL)**	34.04±3.70	36.13±2.78	380.62±73.42	330.36±50.23
**anti-TG (IU/mL)**	15.32±1.97	15.50±1.90	32.97±4.27	36.38±7.70
**Current dose of L-thyroxine (μg/day)**	None	110.95±5.25	88.46±1.55	None
**GRIN2B ng/ml**	6.234±0.729	1.7915±0.36 (p<0.001)	9.866±0.943 (p<0.001)	6.062±06 (0.4487)

Findings are exhibited as a mean±standard deviation; p – Student’s t test compared the controls and study group.

The diagnosis of hypothyroidism was reached using recommendations of the American Association of Clinical Endocrinologists established in 2012. The diagnosis of AIT considered corresponding clinical characteristics, results of thyroid sonogram (reduced echogenicity) and detected circulating antibodies to thyroid antigens, anti-TPO, and anti-TG [20].

Blood specimens from the patients and control subjects were collected in the morning (8 to 10 AM), following an overnight fasting period. For each individual enrolled in the study, levels of thyroxine (fT4, normal range 6.0–13.0 pmol/L for masculine patients and 7.0–13.5 pmol/L for feminine patients), thyroid-stimulating hormone (TSH, reference range 0.3–4.0 mIU/mL), anti-thyroid peroxidase (anti-TPO, reference range 0–30 IU/mL) and anti-thyroglobulin (anti-TG, reference range 0–65 IU/mL) were defined using STAT FAX303/Plus analyzer (Awareness Technology Inc, USA). Study exclusion criteria were as follows: less than 18 years of age, malignancy, inflammation due to rheumatic diseases or acute/chronic infection, diabetes, vascular diseases, chronic liver or kidney diseases, and pregnancy. Individuals administered drugs that could inhibit thyroid function were also removed from the study.

### Enzyme-linked immunosorbent assay (ELISA)

GRIN2B levels in the sera of the patients and healthy subjects were assessed by enzyme-linked immunosorbent assay, which is highly sensitive to Human GRIN2B (Glutamate Receptor, Ionotropic, N-Methyl-D-Aspartate 2B) ELISA Kit (Elabscience^®^, United States, Catalog No E-EL-H1631).

### Genotyping of the glutamate ionotropic receptor NMDA type subunit 1, GRIN1 (rs4880213) gene polymorphism

#### DNA isolation

Venous blood from patients was collected in a sterile Vacutainer and stabilized with K2EDTA. Total DNA was isolated from peripheral blood using the PREP-RAPID-GENETICS DNA Extraction Kit (DNA-TECHNOLOGY, Russian Federation), following the manufacturer’s instructions.

#### DNA amplification and genotyping

All specimens were genotyped applying TaqMan probes and TaqMan Genotyping Master Mix (4371355) on CFX96™ Real-Time PCR Detection System (Bio-Rad Laboratories, Inc., USA). Polymerase chain reaction (PCR) for TaqMan genotyping was conducted, adhering to the requirements enclosed in the kit (Applied Biosystems, USA). TaqMan Genotyping Master Mix contains DNA polymerase AmpliTaq Gold^®^, dNTPs, reference dye ROX™, and the composition of buffers. TaqMan probes are target-specific oligonucleotides with reporter dyes labeled at the 5’ end of each probe: (VIC^®^ dye at the 5’ end of the Allele 1 probe and 6 FAM™ dye at the 5’ end of the Allele 2 probe), and a non-fluorescent quencher (NFQ) labeled at the 3’ end of the probe. Genomic DNA was intensified in a 10 μL reaction mix comprising genomic DNA, forward and reverse primers, fluorescent probes, and TaqMan Genotyping Master Mix. Genotyping of the specimens was conducted on the CFX-Manager™ software applying the technique of allele discrimination based on the magnitude of relative fluorescence units (RFU).

### Statistical analysis

To determine the distinction among groups, we used Student’s t-test, ANOVA, Pearson’s χ^2^ test, odds ratio test, relative odds ratio test, and equality 0 correlation test. We calculated the odds ratio and 95% confidence interval (CI) using binary logistic regression. P values <0.05 were considered a statistically relevant difference when comparing two groups.

## Results

Allele and genotype frequencies for NMDA rs4880213 in patients with thyroid disorders and the controls were analyzed. The relative frequency of these gene polymorphic variants did not differ within both groups (Table 2).

**Table 2.** Distribution of rs4880213 polymorphism in the surveyed population.

**rs4880213** **n=178 (%)**	**Study group** **n=153 (86.96%)**	**Control group** **n=25 (14.04%)**	**OR** **[95% CI]**	**χ**^2^ **(p)**
**CC**	50 (32.68%)	6 (24%)	1.5337 [0.545; 4.99]	**χ**^2^=0.4 (p=0.5259)
**CT**	70 (45.75%)	15 (60%)	0.56 [0.212; 1.44]	**χ**^2^=1.22 (p=0.269)
**TT**	33 (21.57%)	4 (16%)	1.44 [0.442; 6.1738]	**χ**^2^=0.1372 (p=0.711)
**χ^2^** **P**	**χ**^2^=13.451 (p=0.0012)	**χ**^2^=8.24 (p=0.01624)		
**C allele**	170 (55.56%)	27 (54%)	1.065 [0.556; 2.026]	0.003 (p=0.959)
**T allele**	136 (44.44%)	23 (46%)		

OR – odds ratio; n – total number.

The odds ratio (OR) test indicates that the distribution of genotypes CC, CT, and TT presents no difference between the study and control groups. The CT genotype of the NMDA gene (rs4880213) was prevalent in the population under survey: 45.75% in the study group *vs.* 60% in the control group. The homozygous CC genotype was 32.68% *vs.* 24%, respectively. Finally, the TT genotype was 21.57% in the study group *vs.* 16% in the control group (p>0.05).

The C allele of the NMDA gene was more frequent than the T allele among thyroid patients: 55.56% carried the C allele *vs.* 54% in the controls (p> 0.05); the respective frequencies for the T allele were 44.44% *vs.* 46% (p> 0.05). The distribution of rs4880213 variations in the patients considering different thyroid pathology is shown in Table 3. Distribution of rs4880213 variations among patients considering different types of thyroid pathology demonstrated no pronounced difference in the relative frequency of NMDA polymorphic variants among the patients of PO, AIT with hypothyroidism, and AIT groups (Table 3).

**Table 3.** Distribution of rs4880213 genotypes in the study group depending on the type of thyroid pathology and control group.

**rs4880213**	**Control group** **n=25** **(14.04%)**	**Study Group** **n=153** **(85.96%)**	**Group 1 PO** **n=16 (10.46%)**	**Group 2** **AIT with** **hypothyroidism** **n=65** **(42.48%)**	**Group 3** **AIT** **n=72** **(47.06%)**	**χ**^2^ **P**	**Total** **n=178** **(%)**	**OR** **[95% CI]**
**CC**	6 (24%)	50 (32.68%)	4 (25%)	22 (33.85%)	24 (33.33%)	**χ**^2^=1.24 (0.7425)	56 (31.46%)	0.634 [0.183; 1.938]
**CT**	15 (60%)	70 (45.75%)	7 (43.75%)	30 (46.15%)	33 (45.83%)	**χ**^2^=1.779 (0.6196)	85 (47.75%)	1.762 [0.641; 5.029]
**TT**	4 (16%)	33 (21.57%)	5 (31.25%)	13 (20%)	15 (20.83%)	**χ**^2^=1.4362 (0.6971)	37 (20.79%)	0.726 [0.157; 2.643]
**χ^2^** **P**	**χ**^2^=8.24 (0.01624)	**χ**^2^=13.451 (0.0012)	**χ**^2^=0.875 (0.6456)	**χ**^2^=6.677 (0.0355)	**χ**^2^=6.75 (0.0342)			

In subsequent experiments, we determined GRIN2B level in blood serum (Table 1). Our results showed a pronounced reduction in serum GRIN2B in patients with postoperative hypothyroidism by 3.45-fold compared with the controls. Even so, a probable increase in GRIN2B levels by 1.58-fold compared with controls was observed along with AIT-induced hypothyroidism. In addition, our study identified that in patients with AIT without hypothyroidism, the level of GRIN2B was not markedly different from the controls (Table 1).

In a more detailed analysis of GRIN2B levels in patients of different groups, the indicators significantly differed depending on thyroid pathology (Table 4). At the same time, no significant difference between GRIN2B content was found (Table 4) between carriers of different genotypes of the rs4880213 gene. Correlation analysis of the association between GRIN2B and anti-TPO and anti-TG was performed (Table 5). The observed dependence of anti-TPO from GRIN2B is described by a linear regression equation:



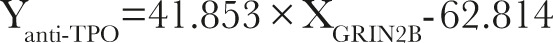



**Table 4.** GRIN 2B in patients with different thyroid pathology depending on the rs4880213 genotype.

**rs4880213 (M±m)**						
**rs4880213**	**CC**	**CT**	**TT**	**P5**	**P6**	**P7**
**Control group, n=25**	6.11±0.91 (6)	6.28±0.717 (15)	6.213±0.43 (4)	>0.05	>0.05	>0.05
**Study group, n=153**	7.45±3.51 (50)	7.04±2.89 (70)	6.01±4.11 (33)	>0.05	>0.05	>0.05
**P1**	>0.05	>0.05	>0.05			
**PO n=16**	1.68±0.59 (4)	1.95±0.334 (7)	1.702±0.22 (5)	>0.05	>0.05	>0.05
**P1**	0.00019	<0.0001	<0.0001			
**P2**	<0.0001	<0.0001	<0.0001			
**AIT with hypothyroidism n=65**	10.1±1.047 (22)	9.56±0.95 (30)	10.3±0.428 (13)	>0.05	>0.05	<0.05
**P1**	<0.0001	<0.0001	<0.0001			
**P3**	<0.0001	<0.0001	<0.0001			
**AIT n=72**	5.85±0.835 (24)	6.14±0.586 (33)	6.06±0.02 (15)	>0.05	>0.05	>0.05
**P1**	>0.05	>0.05	>0.05			
**P4**	0.00058	<0.0001	<0.0001			

P1 – p-value between the control group and study groups; P2 – p-value between PO and AIT with hypothyroidism groups; P3 – p-value between AIT and AIT with hypothyroidism groups; P4 – p-value between PO and AIT groups; P5 – p-value between AA and AG genotypes; P6 – p-value between AA and GG genotypes; P7 – p-value between AG and GG genotypes.

**Table 5.** The correlation analysis of the association between GRIN2B and anti-TPO and anti-TG.

**Variable**	**Correlation characteristics**		
	**ρ**	**The strength of the association was assessed using the Chaddock scale**	**P**
**GRIN2B – anti-TPO**	**0.635**	**close correlation**	p<0.001 *
**GRIN2B – anti-TG**	**0.527**	**close correlation**	p<0.001 *

* – differences are statistically significant (p<0.05).

**Figure 1.**
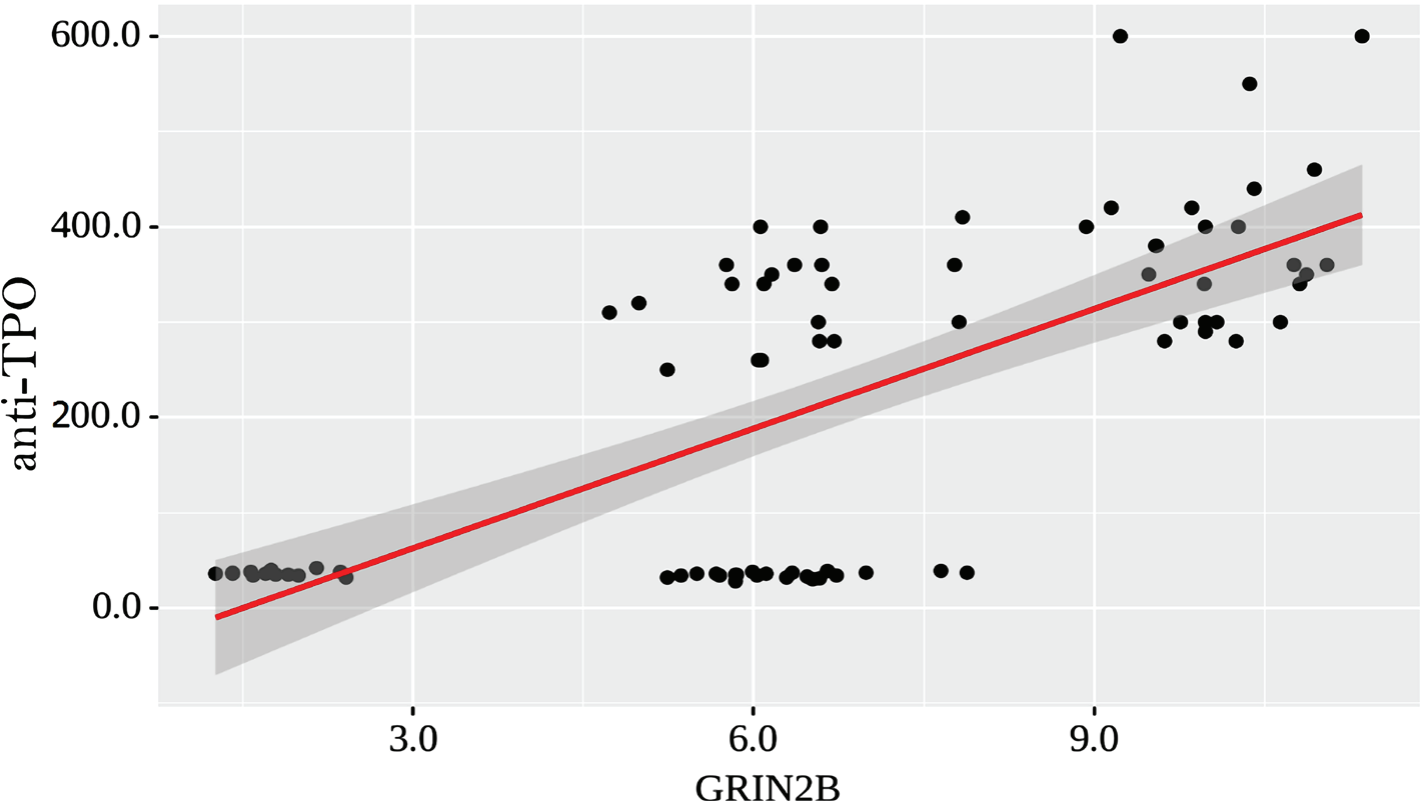
Regression line characterizing the dependence of anti-TPO from GRIN2B.

With 1 increase of GRIN2B, a 41.853 change of anti-TPO should be expected (Figure 1). According to the coefficient of determination R^2^ of the resulting model, 48.9% of the observed variance of anti-TPO was explained.

The observed dependence of anti-TG from GRIN2B is described by a linear regression equation:



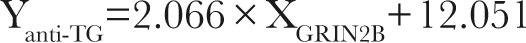



**Figure 2.**
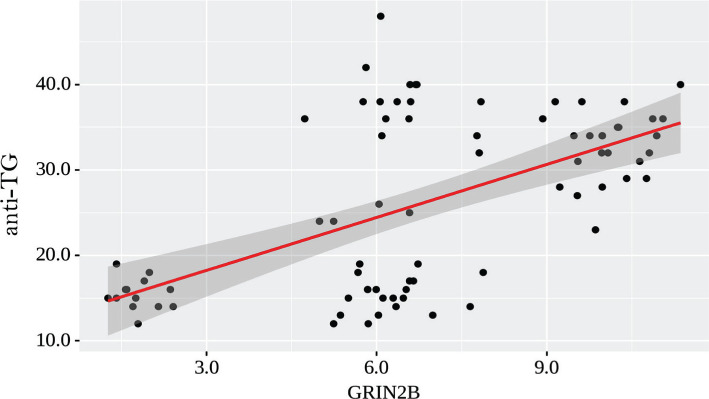
Regression line characterizing the dependence of anti-TG from GRIN2B.

With 1 increase of GRIN2B, a 2.066 change of anti-TG should be expected. According to the coefficient of determination R^2^ of the resulting model, 34.2% of the observed variance of anti-TG were explained (Figure 2).

## Discussion

The discovery of the role of genetic factors in the development of widespread multifactorial diseases is of great importance and remains currently one of the main issues of biomedical research. In the present work, we studied the NMDA gene polymorphism at the rs4880213 locus of subjects with various thyroid gland disorders in the Bukovinian region of Ukraine. All NMDARs are likely to function as heteromeric parts comprising several NR1 subunits and no less than one NR2 type [21]. In situ hybridization researchers have found that mRNAs for NMDAR subunits are allocated across the brain, and the expression schemes of such mRNAs are altered during development [22]. Cases of disruptive behavior and depression were notably less in subjects who had the T/T GRIN1 genotype (SNP rs4880213) than in the patients of the other two groups. Francesco *et al.* [23] claimed that the T/T SNP GRIN1 rs4880213 genotype was related to decreased intracortical inhibition, increased glutamatergic excitation, and heightened NMDAR glutamate function. In our study, the CT genotype of the NMDA gene (rs4880213) was predominant in the surveyed population. The homozygous CC genotype was the second most common. The TT genotype was the least common: 21.57% in the group under study *vs.* 16% in the controls. The C allele of the NMDA gene was more frequent compared to the T allele in the thyroid patients: among the thyroid pathology patients, 55.56% carried the C allele *vs.* 54% in the control group (p> 0.05); the respective frequencies for T allele were 44.44% *vs.* 46% (p> 0.05). The T and C alleles’ population frequency in this study corresponds to the average for European populations (https://cutt.ly/Vmhz2I2). Another study also showed that the subunits including GRIN1 and GRIN2B NMDAR are required to regulate cortical excitability and human cortical plasticity [23].

In our study, GRIN2B levels were significantly different in patients of different groups depending on thyroid pathology: a pronounced decrease in serum GRIN2B in patients experiencing PO 3.45 times compared to the control group. On the other hand, there was a probable increase in GRIN2B levels in patients with AIT-induced hypothyroidism by 1.58 times compared with controls. At the same time, in patients with AIT without hypothyroidism, the level of GRIN2B was not significantly different from the control group. Furthermore, Rossi *et al.* [24] also detected that the C rs4880213 allele is connected with decreased NMDAR-mediated cortical excitability. Noteworthy, some evidence reported NMDAR dysfunction related to depression syndrome [3]. Consequently, the homozygosity of the GRIN1 rs4880213 T allele can enhance the function of NMDAR glutamate and regulate mood compared to other alleles. However, since the influence of GRIN1 rs4880213 SNP on the NMDAR function has not yet been expounded at the molecular or meta-level, it is necessary to study how SNP GRIN1 rs4880213 influences NMDAR function.

Distribution of rs4880213 variations among patients considering different types of thyroid disorders demonstrated no pronounced difference in the relative frequency of NMDA polymorphic variants among the patients of different groups, namely PO, AIT with hypothyroidism, and AIT. In addition, we did not find significant differences in GRIN2B levels between the carriers of different genotypes of the rs4880213 gene.

One of the communication mechanisms between NMDA receptors and neurological complications of hypothyroidism may be realized through kynurenine metabolism. The metabolism of essential amino acids, L-tryptophan, along the kynurenine pathway, gives some neuroactive compounds, which comprise neuroprotective kynurenic acid and neurotoxic 3-hydroxykynurenine and quinolinic acid [25]. Kynurenic acid exhibits specific biological properties. It is a well-defined endogenous antagonist of glutamatergic receptors, with a considerably high affinity for the glycine site of NMDA receptor complex, an antagonist of α7 nicotinic receptors, and ligand of aryl hydrocarbon receptors, and of orphan receptors GPR35 [26].

Correlation analysis of GRIN2B levels and the levels of TSH, T4, anti-Tg, and anti-TPO antibodies demonstrated a strong correlation (r=0.635) between GRIN2B and anti-TPO levels (p <0.001), a notable direct correlation (r=0.527) between GRIN2B and anti-TG levels in the blood (p<0.001).

Physiologically deficient concentrations of kynurenic acid can change glutamate release, as displayed in rat striatum or hippocampus [27]. Synthesis of kynurenic acid occurs in the periphery and the brain due to an irreversible transamination of L-kynurenine catalyzed by kynurenine aminotransferases (KATs I-III). Roughly 75% of brain kynurenic acid is synthesized in situ by astrocytic KAT II (α-aminoadipate transferase; AADAT). Various signal transduction pathways related to the cellular response to stress, inflammatory processes, damage, and several pharmacological agents, can modulate kynurenic acid formation [28]. Research conducted on humans and animals provided compelling proof that deficiency of kynurenic acid may induce neuronal loss [29]. On the other hand, increased brain kynurenic acid can cause cognitive impairments due to decreased signaling through NMDA and α7 nicotinic receptors, which are of great importance for learning and memory [30].

## Conclusion

In our study, GRIN2B levels were significantly decreased in patients with postoperative hypothyroidism (3.45 times). Moreover, there was a probable increase in GRIN2B levels in patients with AIT-induced hypothyroidism by 1.58 times compared with controls. We did not find a significant difference in the GRIN2B levels between the carriers of different genotypes of the rs4880213 gene. Our study showed close correlation (r=0.635) between GRIN2B and anti-TPO levels (p <0.001), a significant direct correlation (r=0.527) between GRIN2B and anti-TG levels in the blood (p<0.001).

## Acknowledgments

### Conflict of interest

The authors declare no conflict of interest.

### Ethical approval

This study was approved by the Ethics Committee of the Bukovinian State Medical University, Ivan Horbachevsky Ternopil National Medical University, and Chernivtsi Regional Endocrinology Center, Ukraine (approval ID: 11-07.11.2017). Our study was conducted according to the Declaration of Helsinki adopted in 1975 and revised in 2008, and the ethical principles were entirely respected.

### Consent to participate

Written informed consent was obtained from the participants.

### Data availability

The data of this study is available by request.

### Authorship

IIK and AMK contributed to conceptualizing, methodology, writing the original draft, editing and data analysis, review and approval of the final version. LBP contributed to data collection, review of the draft manuscript, and approval of the final version. All authors had full access to all the data in the study and had final responsibility for the decision to submit for publication.
